# CAR-Treg cell therapies and their future potential in treating ocular autoimmune conditions

**DOI:** 10.3389/fopht.2023.1184937

**Published:** 2023-04-18

**Authors:** Alan R. Abraham, Panayiotis Maghsoudlou, David A. Copland, Lindsay B. Nicholson, Andrew D. Dick

**Affiliations:** ^1^ Ophthalmology Research Group, Academic Unit of Ophthalmology, Translational Health Sciences, University of Bristol, Bristol, United Kingdom; ^2^ University of Bath, Bath, United Kingdom; ^3^ UCL-Institute of Ophthalmology, University College London, London, United Kingdom

**Keywords:** cell therapy, CAR-Treg, Treg, ophthalmology (MeSH), ocular gene therapy, autoimmune diseases

## Abstract

Ophthalmic autoimmune and autoinflammatory conditions cause significant visual morbidity and require complex medical treatment complicated by significant side effects and lack of specificity. Regulatory T cells (Tregs) have key roles in immune homeostasis and in the resolution of immune responses. Polyclonal Treg therapy has shown efficacy in treating autoimmune disease. Genetic engineering approaches to produce antigen-specific Treg therapy has the potential for enhanced treatment responses and fewer systemic side effects. Cell therapy using chimeric antigen receptor modified T cell (CAR-T) therapy, has had significant success in treating haematological malignancies. By modifying Tregs specifically, a CAR-Treg approach has been efficacious in preclinical models of autoimmune conditions leading to current phase 1-2 clinical trials. This review summarises CAR structure and design, Treg cellular biology, developments in CAR-Treg therapies, and discusses future strategies to apply CAR-Treg therapy in the treatment of ophthalmic conditions.

## Introduction

Chimeric antigen receptor (CAR) T cell therapy has had remarkable success in the therapy of haematological malignancies, leading to relapse-free treatment response in patients with previously treatment-resistant leukaemia and lymphoma (1). CAR-T therapy redirects a patient’s own T cell immune responses to target and eliminate cancer cells, as seen with CD19-targeted CAR T cells that eliminate a patient’s B cell compartment. The first CAR-T cell therapy received US FDA approval in 2017 and since then the field has expanded substantially with hundreds of ongoing clinical trials (2, 3).

CAR-T therapy is not without risk of serious adverse events, such as B cell compartment elimination in CD19-directed CAR-T therapy, cytokine release syndrome (CRS), and immune effector cell-associated neurotoxicity syndrome (ICANS) (4). CRS occurs secondary to systemic cytokine release leading to high fever, hypoxia, hypotension, and neurologic symptoms (1, 4). ICANS manifests most commonly with acute delirium but can also be life-threatening secondary to cerebral oedema and seizures (1, 4).

Although current CAR-T therapies that have been released to market have exclusively been used in cancer treatment, there is increasing research interest in applying CAR technology in the treatment of immune mediated inflammatory disorders (IMIDs) (5).

## Tregs and immune tolerance

Tregs, a regulatory subset of CD4+ T-cells, comprise approximately 10% of the total CD4+ T cell compartment, and are characterised by the expression of the cell surface high affinity IL-2 receptor CD25 and the transcription factor FoxP3+ (6). Thymic-derived ‘tTregs’ develop through central tolerance mechanisms (7). In contrast, peripheral ‘pTregs’ are induced following the exposure of effector CD4+ T cells to conditions including the presence of IL-2 and TGF-beta (7). tTregs have T-cell receptor (TCR) specificity for autoantigens imprinted in the thymus and have a key role in immune self-tolerance. pTreg promote immune homeostasis, often at barrier sites, with their dysregulation being associated with the development IMIDs, including rheumatoid arthritis (RA), systemic lupus erythematosus (SLE), multiple sclerosis (MS), and inflammatory bowel disease (IBD) (6). The key role of Tregs in immune regulation is evidenced by the disease polyendocrinopathy enteropathy X-linked (IPEX) syndrome occurring in males with functional variants of the *FOXP3* gene (8), and a similar lethal autoimmunity that is recapitulated in the scurfy mice strain which carry pathogenic *FOXP3* variants (9).

**Figure 1:**
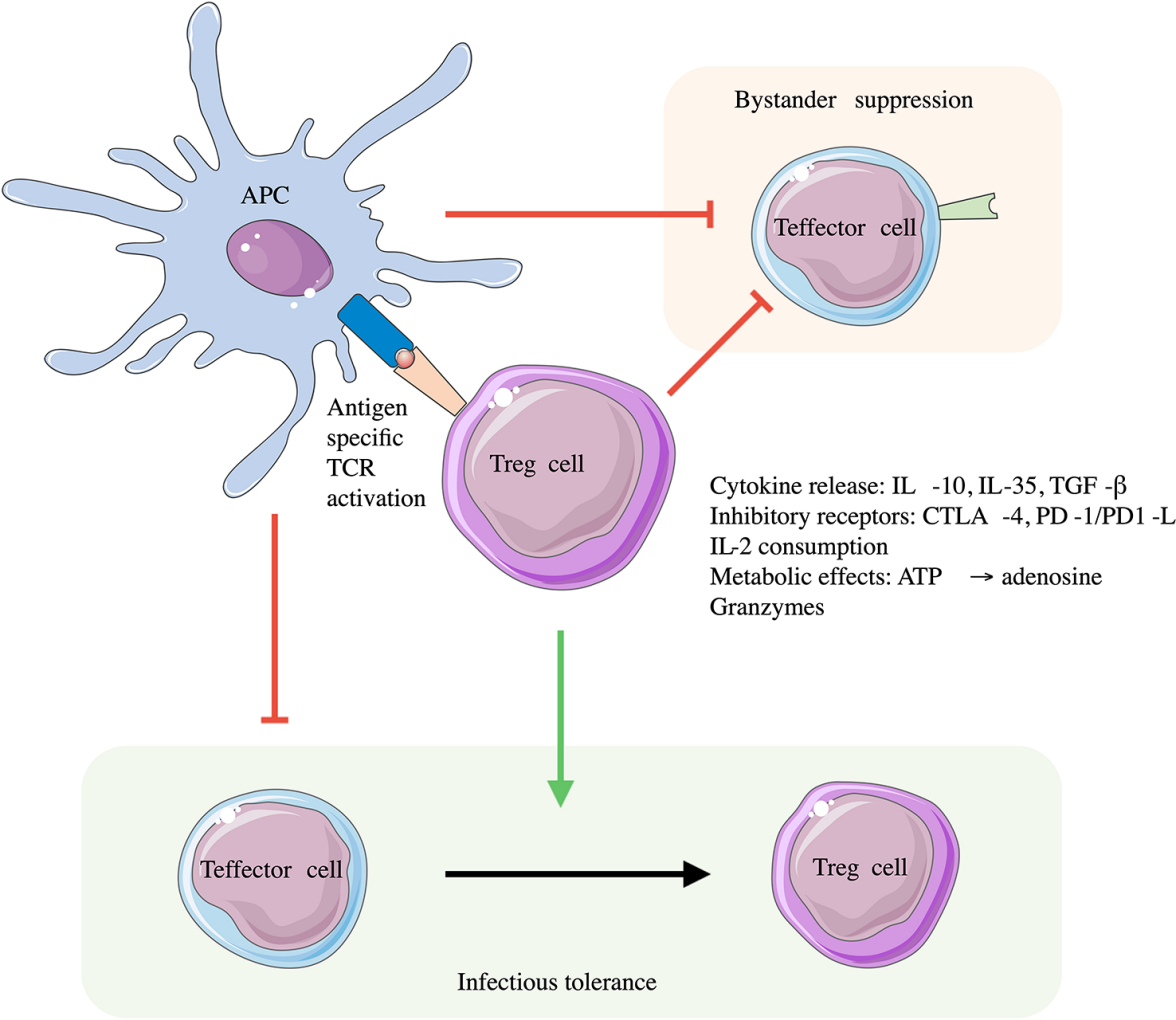
Roles of Tregs in immune suppression. Tregs are activated through their antigen specific TCR. Engagement with antigen presenting cells (APCs) *via* the antigen specific MHC-peptide and Treg TCR interaction leads to downregulation of APC immune stimulatory activity and inflammatory cytokine production. This induces a tolerogenic phenotype in APCs suppressing Teffector cell activity. Once activated Tregs can regulate immune responses through both contact and non-contact mediated methods which themselves are not antigen specific, leading to bystander suppression of effector T-cells of different TCR specificity, and promoting infectious tolerance with the conversion of Teffector cells to Tregs. Adapted from (11).

Tregs express a TCR that directs their tissue specificity and activation state (10). However, many Treg suppressive functions are not antigen specific allowing suppression of effector cells of multiple specificities (termed bystander suppression) (11). This further leads to local alterations in the immune environment, which can be long lived by promoting development of other regulatory immune cell populations e.g., pTregs, Tr1 cells; a process called infectious tolerance (11) **(** **Figure 1** **).** Tregs produce antigen specific suppression *via* their TCRs interacting with cognate antigen presented *via* MHC on dendritic cells (DC) and CD4+ T cells (12). Once activated, Tregs have several antigen non-specific mechanisms of action, these include but are not limited to: release of inhibitory cytokines (IL-10, IL-35, TGF-β) (6, 12); consumption of IL-2 inhibiting T-effector cell differentiation and function (13); expression of PD-1 and PD-L1 inhibiting T-cell proliferation; and CD39/73 expression leading to pro-inflammatory ATP degradation to adenosine thus exerting an anti-inflammatory effect (14). These antigen independent effects also promote a tolerogenic phenotype in DCs which function with activated Tregs to further induce regulatory T-cells, and suppress effector T-cells (10). Other mechanisms identified include the release of granzyme B, which lead to direct killing of both APCs and CD4+/CD8+ effector cells; upregulation of CTLA-4 and enhanced DC indoleamine 2,3-dioxygenase (IDO) activity promoting Teffector cell anergy. The functional roles and specialisation of different subtypes of Treg are reviewed in detail in (6, 10, 12).

Bystander suppression and infectious tolerance leading to both acute immune suppressive effects and long-term shifts in the local immune environment has sustained efforts to use Tregs as a cell therapy approach (5, 15). Adoptive cell transfer of CD4+ CD25+ T-cells was initially trialled in athymic nude mice (with concomitant loss of central tolerance mechanisms) successfully suppressing self-reactive T-cells. This approach has been trialled in patients with IMIDs including Type I Diabetes (16, 17), and ulcerative colitis (18), and as an adjuvant to immune suppression in the context of solid organ transplantation (19, 20). Although partially effective, use of polyclonal Treg therapy entails the risk of generalised immunosuppression and loss of tumour immunosurveillance (21). Antigen specific approaches to Treg therapy have also demonstrated greater efficacy in therapeutic responses in pre-clinical studies, with a lower risk of off-target side effects (11, 22).

Generating sufficient antigen specific Tregs for cell therapies from naturally occurring populations is complicated by low starting numbers of Tregs with the desired TCR specificity (11). Alternative approaches have included the use of engineered receptors to re-direct the specificity of a starting population of Tregs, or the conversion of antigen specific effector T-cells into Tregs through enforced FOXP3 expression (23). For all strategies to utilise natural Tregs or converted effector cells, there is a significant concern of phenotype instability, leading to an antigen-specific Teffector cell therapy being generated with the opposite intended effect for Treg therapy. This appears to be modulated by TCR signalling (24) and the inflammatory microenvironment (25).

## Chimeric antigen receptors: structure and production

**Figure 2:**
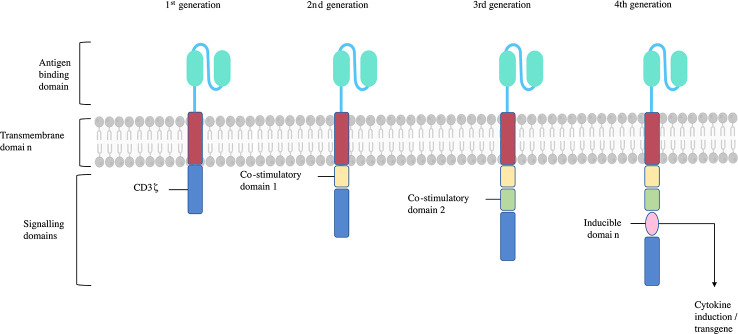
CAR structure and generations. CARs consist of an antigen binding domain, hinge, transmembrane domain, and single or multiple signalling domains. The 1^st^ generation CARs sole signalling domain was the CD3ζ subunit, activating their T-cell upon CAR binding its antigen target. 2^nd^ and 3^rd^ generation CARs include one or two additional signalling domains respectively. These are typically CD28 and/or 4-1BB which help stabilise and enhance T-cell function. Subsequent CAR generations include more signalling domains or further subunits endowing additional functions such as cytokine or chemokine production, inducible ‘suicide’ constructs, or transcription factors. Adapted from (26).

CARs offer a different approach to re-direct Treg specificity. A CAR in its most essential form consists of an extracellular antigen binding domain, and an intracellular T-cell signalling domain (23) (**Figure 2**). There are several advantages of CARs over engineered TCRs, including their ability to exploit mechanisms of target recognition without the requirement of MHC co-presentation. Consequently, CARs can bind a much wider repertoire of antigenic targets than a natural or engineered TCR, including whole proteins, and this can be further modified based on the antigen binding domain (11, 23).

The antigen binding domain of a CAR confers the antigen specificity that directs the cell to a specific target. These have typically derived from connected variable fragment heavy (VH) and light (V_L_) domains monoclonal IgG derived antibodies producing a single-chain variable fragment (scFv) (27). scFvs usually target extracellular surface expressed antigens with their target recognition plus binding leading to MHC independent T-cell activation through the CAR CD3ζ intracellular signalling domain. Alternatives to scFvs have been investigated as well, such as use of the V_H_ domains of camelid antibodies, natural ligands, and other artificial protein binding constructs (28). The hinge or spacer region links the antigen binding domain to the transmembrane domain. The flexibility of the hinge region affects the ability of the antigen binding domain to access the antigen epitope it binds to (27). The intracellular signalling domains of CARs first used CD3ζ in isolation in first generation CARs. When activated by target recognition by the antigen binding domain, this domain initiates TCR like signalling (28). Subsequent generations of CAR are characterised by additional co-stimulatory domains, whether one or two additional domains (second and third generation CARs respectively), or the release of cytokines on activation (fourth generation CARs) (23). Further modifications to CARs can enhance the phenotype stability of the Treg they are added to by, for example, constitutive FoxP3 or IL-2 production which are intended to help reduce the risk of Tregs developing T effector function (23, 29).

Processes to generate CAR-Tregs typically use lentiviral transfection of already isolated Tregs (5, 23). Other methods to insert CAR transgenes have also been explored including non-viral vectors (liposomes) or electroporation, and the use of CRISPR/TALEN based genetic engineering approaches (15). Inducing ectopic FoxP3 expression has also been explored, but this does not appear to be sufficient to induce a functional Treg phenotype when starting with CD4+ cells, given the limited efficacy of FoxP3 transfected Tregs *in vivo* (30, 31).

Currently, to produce CAR-Tregs for clinical use, CD4+ T-cells must first be harvested and isolated from the patients receiving the therapy. The cells are then further purified and cultured to produce Tregs before transfection with a viral vector. The CAR-Treg cells produced are then re-administered to the patient. This process requires local expertise, facility infrastructure and monitoring requirements, leading to high costs to the using therapeutic CAR-Tregs (32). Future developments may reduce the associated costs of delivering CAR-T related therapies, through broadening indications across IMIDs requiring focus on improving manufacturing pipeline, and development of off-the-shelf CAR-T products such as ‘universal’ CARs or the use of allogeneic ‘universal’ starting blood products (32).

## CAR-T therapy in treatment of autoimmune conditions

Applying CAR-T therapy to treat IMIDs has taken several forms. This has including use of CAR-T therapies which direct immune responses against a specific target, like the use of CAR-T therapy in cancers. Anti-B cell CAR T therapy has been applied successfully to treat patients with SLE following successful proof of concept in mouse models of SLE (33, 34). This approach is also being explored in other diseases related to autoantibody production such as myasthenia gravis (35). More fine-tuned targeted elimination of specific subsets of autoantibody producing B-cells has also been explored as seen with chimeric autoantigen receptor T-cells (CAAR T therapy) which has demonstrated efficacy in models of pemphigus vulgaris and factor VIII deficiency (36, 37). This approach may reduce systemic side effects such as generalised immunosuppression that occur with complete elimination of the B cell compartment. Many IMIDs are also driven by T-cell responses orchestrating local immune dysregulation, with a lack of clear autoantigen driven targets for elimination using a CAR-T effector cell approach.

Using CARs to target Tregs offers the prospect of more long-lived immune regulation with fewer risks of adverse events such as CRS seen with conventional CAR-T therapy. Currently studies are applying CAR-Treg therapy to prevent or treat transplant rejection and Graft versus Host Disease (GvHD) with recruitment of patients started since 2022 in phase I/II clinical trials for a CAR specific for HLA-A2 in kidney transplant patients (5).

Pre-clinical evidence has also accumulated using a CAR-Treg approach in several models of autoimmune diseases. The 2,4,6-trinitrobenzene sulfonic acid induced colitis model was treated successfully with CAR- 2,4,6-trinitrophenol Tregs both in managing disease and preventing development of the colitis model (38). CAR Tregs specific for carcinoembryonic antigen (CEA) have also been shown to localise to the colon, and prevent development of colitis (39). In Type 1 Diabetes (T1D) the use of islet specific Treg adoptive cell therapy was able to stabilise and reverse diabetes in a mouse model (40) supporting antigen specific Treg therapy. A CAR-Treg developed with specificity for a pancreatic beta cell epitope (GAD65) demonstrated stable homing to pancreatic cells in a humanized mouse model of T1D, with evidence for improved glycaemic control following treatment reported (41). For RA, a CAR Tregs targeting type II collagen have partially reversed collagen induced arthritis (CIA), a mouse model of RA (42). A CAR targeting citrullinated vimentin (CV), a post transcriptionally modified protein highly specific for synovial inflammation in active RA, has demonstrated activity against *in-vitro* to CV in patient synovial fluid and CV expressing cells when transduced into Tregs (43). The experimental autoimmune encephalomyelitis (EAE) murine model of MS can be modulated using adoptively transferred CAR-Tregs specific for myelin oligodendrocyte glycoprotein (MOG). The transferred MOG CAR-Tregs successfully localised to the brain following intranasal delivery leading to reduced disease activity scores in EAE induced mice (44). Myelin basic protein (MBP) TCR transgenic Tregs both successfully localised to brain and spinal cord tissues and reduced EAE disease activity in mice (45). For conditions with disease pathology related to antibody production such as SLE or GvHD there may also be a role for use of CAR-Tregs in directly suppressing B-cell driven antibody production, as seen with a CD-19 targeting CAR-Treg in pre-clinical models of GvHD (46, 47). These findings provide a proof of concept to further translating CAR-Treg therapy towards treating human disease.

## Applying CAR-Treg therapy in ophthalmic care

Like other IMIDs, Tregs also influence regulation of ocular IMIDs. For example, Tregs are upregulated in the disease resolution phase of the animal models of uveitis, experimental autoimmune uveoretinitis (EAU), with concomitant increase in IL-10 and TGF-Beta levels measured (48) There is also reported variation in the ratio of Tregs to Teffector cells in patients with uveitis during active disease (49). There is competing evidence regarding whether functionality of Tregs during active inflammation in EAU is impaired or not (48, 50). Patients with uveitis also show conflicting evidence of Treg dysfunction with stable suppressive Tregs detected in aqueous samples of patients with active uveitis versus dysfunctional Tregs detected in peripheral blood samples (51). Nevertheless, the role of Tregs is evident with their depletion in mice leading to more severe manifestations of EAU (52). This effect of Tregs is also seen in ocular surface conditions, with exacerbation of a mouse model of ocular Sjögren’s following Treg depletion (53) and adoptive transfer of Tregs successfully suppressing ocular surface inflammation (54).

Ocular IMIDs are an attractive target for CAR-Treg therapy. Diseases such as posterior uveitis and corneal IMIDs (e.g. GvHD (55), peripheral ulcerative keratitis (PUK) (56)) present significant risks of sight loss and are difficult to treat effectively. Adapting CAR-Treg therapy has also been applied with success in transplant rejection (20); this can be extended to expanding access to corneal grafts in ophthalmology (57), offering a novel approach to managing higher risk indications for corneal transplantation. There is also the prospect of applying CAR-Treg therapy as an adjuvant to enhance the effect of existing ocular gene therapies (58, 59). Given the accessible nature of the eye, it may also be possible to directly deliver CAR-Treg therapies into target tissues using subconjunctival, intracameral, intravitreal, or subretinal approaches (60). These delivery routes may introduce constraints on maximum therapeutic dose able to be delivered compared with intravenous transfusion, in addition to introducing procedure specific risks such as haemorrhage, endophthalmitis, or the development of retinal tears (60). Nevertheless, intravitreal delivery of polyclonal Tregs has previously demonstrated efficacy in treating a uveitis model in mice (61).

Current treatment modalities for non-infectious uveitis employ tapering steroid therapies, alongside systemic immunosuppression to reduce uveitis flares and minimise the steroid load (62, 63). Immunotherapies have been effective in reducing the ongoing need for higher doses of steroids in a proportion of patients with uveitis, however, there remains a large patient cohort who experience uveitis relapses despite high levels of immunosuppression (64). Corneal surface related IMIDs such as PUK Sjögren’s syndrome or GvHD are also managed with escalating steroid and immunotherapy regimens in addition to ocular surface protection (55, 56, 65). The use of CAR-Treg therapies offers the prospect of an alternative therapy that may lead to long-lasting immunosuppression with a localised treatment effect and help reduce the ongoing need for higher doses of steroid therapies which themselves cause side effects.

Corneal transplants, including partial- or full-thickness grafts typically remain stable with topical steroid therapy alone, with often no ongoing need for medication (66). However, in patients with higher-risk grafts, ongoing use of higher dose steroid or immunotherapy regimens may be needed to prevent acute or chronic graft rejection (57). Further, patients with long-standing grafts continue to experience acute and chronic rejection, which can lead to the need for repeat corneal transplantation with a higher risk of repeated rejection (67). CAR-Treg therapy in these patients may encourage peripheral tolerance mechanisms that reduce or reverse the risk of rejection. It is also possible the use of CAR-Treg therapy at the time of surgery could enhance engraftment and long-term outcomes. This may also expand the indications for corneal grafts to include patients with ongoing corneal inflammatory diseases such as PUK or other ocular surface diseases which currently limit or contraindicate the use of corneal grafts (57).

Ocular gene therapies, such as Luxturna, have helped treat inherited retinal diseases with significant impact (58, 59). Although effective in providing visual acuity improvements, ocular inflammation, and immune responses to the gene therapy (both the viral vector and the transgene product) can lead to reductions in final visual outcomes, or treatment failures (68). In addition, repeat treatment may be limited by the development of immune response against the therapy (68). This may also present a significant barrier to future use of further gene therapy products for different diseases; for example if multiple ocular diseases are amenable to viral vector delivered gene therapies, there is the prospect of recurrent immune responses triggered by repeated use of immunologically cross-reactive AAV capsids for different gene therapy products (69). Given the high costs of in current gene therapies (70, 71), which may limit access to one attempt at treatment, any enhancement of therapeutic success modifying immune responses will bring will be highly cost efficient. CAR-Treg therapy in this context may be delivered at the time of gene therapy and help reduce accompanying immune responses, and perhaps limit future responses to re-treatment. An example of the application of CAR-Treg therapy in gene therapy has been developed in an AAV specific CAR-Treg which helped reduce immune responses directed against both the AAV capsid and the transgene product, thereby providing initial evidence of bystander suppression of the CAR-Treg (72).

## Challenges to translating CAR-Treg therapy

To realise the potential of CAR-Treg cell therapies in ophthalmic care, barriers to their safe translation will need to be overcome. The use of antigen-specific CAR-Treg therapy helps address concerns of the development of generalised immunosuppression from polyclonal Treg therapies, with the ensuing loss of tumour surveillance or risk of viral infection (21). Other challenges include improving stability and maintaining Treg functionality in CAR-Tregs – Tregs exposed to inflammatory environments can switch to an effector phenotype. This could lead to the opposite intended effect with CAR-Tregs with immune responses upregulated at the site of disease (24, 25). In addition, there is also the requirement for CAR-Treg treating centres having the capacity to produce CAR-Tregs safely and reproducibly (29).

The hurdles to successful CAR-Treg production arise initially with the design and validation of suitable antigen targets which are expressed specifically in the tissues of interest to treat. The ideal targets for a CAR antigen binding domain having limited expression in other tissue or organ sites (11). These can be antigens associated with disease states or autoantigens expressed by tissues in the affected sites, leveraging the properties of bystander suppression of CAR-Tregs (15). At present there are limited published sequences of eye specific autoantibodies to guide CAR design, hence other techniques to initially characterise antigen binding domains such as the use of hybridoma or phage display might be considered (73). Alternative approaches, targeting antigens associated with disease states and damaged retinal tissue may also prove valuable in eye disease, as achieved elsewhere with CV specific CAR Tregs which are being investigated for RA (43). Ocular IMIDs with pathophysiology related to antibody production such as GvHD (55) or PUK (56) may also benefit from CAR-Treg therapies that directly suppress B-cells (46, 47). Further considerations in CAR design of the intracytoplasmic part of the receptor include the use of co-stimulatory domains and whether these can enhance the immunosuppressive properties of Tregs (29). 2^nd^ generation CD28 CARs appear to have greater stability compared to OX40 2^nd^ generation CAR (74). Further modifications of CAR design have been investigated in promoting safety of the CAR through inclusion of suicide genes, to allow CARTregs to be killed efficiently should adverse proliferation occur (23). Additional modified CARs have been developed which can improve Treg lineage stability of CAR-Tregs by, for example, FoxP3 over-expression, or incorporating gene silencing of IL-17 (23).

Techniques to transfect CARs into Tregs, commonly using viral vectors such as lentivirus or retrovirus, also risk insertional mutagenesis from DNA integration risking neoplasia (28). Alternative approaches leveraging guided transgene delivery may increase the safety profile of CAR-Treg therapies (75). Use of such techniques allows for more physiological expression of the CAR by e.g. linking the transgene to the TCR promotor region when combined with TCR knockout (76). Alternatives to viral vectors are also being explored such as use of liposomes or electroporation however these currently have low transduction efficiency (77). Currently autologous CD4+ cells are the source of Tregs for CAR-Treg therapy, however patients with autoimmune diseases have evidence of deficiencies in Treg function (78, 79). This may affect the efficacy of CAR-Tregs produced using autologous Tregs from these patients as an adoptive cell therapy. Gene editing techniques can be leveraged to remove MHC related genes allowing the use of allogeneic Tregs to produce CAR-Treg therapies (80), this could help resolve barriers related to deficient autologous Treg function in patients with autoimmune conditions.

## Concluding remarks

Future pre-clinical studies are needed to develop proof of concept of use of CAR-Treg therapy of ocular conditions, in parallel with ongoing research to reduce the costs associated with CAR-T therapies, and thereby improve access in future. To deliver this technology initial hurdles need to be overcome. An important step will be the generation of a library of ocular specific antigenic ‘targets’ for CAR antigen binding domains to be developed towards. Further steps toward translation will include demonstration of trafficking of CAR-Tregs into the eye based on the CAR antigen binding domain, and the impact this will have on future treatment modality, for example, intravenous or intravitreal delivery. Applying CAR Treg therapy to ocular conditions may provide a novel disease modifying treatment modality to supplement or enhance current treatment options for severe inflammatory ocular disease.

## Permission to reuse and copyright

Parts of both figures were drawn and adapted using images available from Servier Medical Art. Servier Medical Art by Servier is licensed under a Creative Commons Attribution 3.0 Unported License.

## Author contributions

AA produced the main body of the manuscript including figures. PM and DC contributed to re-drafting the manuscript with suggested edits, re-writes, and formatting. LN and AD both jointly contributed to manuscript as supervisors of AA research fellowship and in providing edits and formatting of the manuscript. All authors contributed to the article and approved the submitted version.
